# Utilizing patient-specific 3D printed kidney surgical guide with realistic phantom for partial nephrectomy

**DOI:** 10.1038/s41598-023-42866-9

**Published:** 2023-09-19

**Authors:** Junhyeok Ock, Taehun Kim, Sungchul On, Sangwook Lee, Yoon Soo Kyung, Namkug Kim

**Affiliations:** 1 Department of Convergence Medicine, Asan Medical Institute of Convergence Science and Technology, Asan Medical Center, University of Ulsan College of Medicine, Seoul, Republic of Korea; 2 Department of Biomedical Engineering, Asan Medical Institute of Convergence Science and Technology, Asan Medical Center, University of Ulsan College of Medicine, Seoul, Republic of Korea; 3 Department of Health Screening and Promotion Center, Asan Medical Center, University of Ulsan College of Medicine, Seoul, Republic of Korea; 4 ANYMEDI Inc., 388-1 Pungnap2-dong, Songpa-gu, Seoul, South Korea; 5 Artificial Intelligence and Robotics Institute, Korea Institute of Science and Technology, Seoul, South Korea

**Keywords:** Cancer, Nephrology, Urology, Biomedical engineering

## Abstract

Partial nephrectomy has been demonstrated to preserve renal function compared with radical nephrectomy. Computed tomography (CT) is used to reveal localized renal cell carcinoma (RCC). However, marking RCC directly and quantitatively on a patient's kidney during an operation is difficult. We fabricated and evaluated a 3D-printed kidney surgical guide (3DP-KSG) with a realistic kidney phantom. The kidney phantoms including parenchyma and three different RCC locations and 3DP-KSG were designed and fabricated based on a patient's CT image. 3DP-KSG was used to insert 16-gauge intravenous catheters into the kidney phantoms, which was scanned by CT. The catheter insertion points and angle were evaluated. The measurement errors of insertion points were 1.597 ± 0.741 mm, and cosine similarity of trajectories was 0.990 ± 0.010. The measurement errors for X-axis, Y-axis, and Z-axis in the insertion point were 0.611 ± 0.855 mm, 0.028 ± 1.001 mm, and − 0.510 ± 0.923 mm. The 3DP-KSG targeted the RCC accurately, quantitatively, and immediately on the surface of the kidney, and no significant difference was shown between the operators. Partial nephrectomy will accurately remove the RCC using 3DP-KSG in the operating room.

## Introduction

Partial nephrectomy has been known to have the same oncologic outcome and is more useful in preserving renal function compared with radical nephrectomy. Recently, outcomes in terms of both survival and morbidity have been found to improve with partial nephrectomy, and an increasing trend toward using partial nephrectomy has been noted in more complex surgical cases^1^. The technique of partial nephrectomy includes the application of vascular clamps on the renal artery and/or renal vein followed by resection of the renal cell mass. The renal vessels and parenchyma are then reapproximated, and the clamps are removed. Adherence to a safe ischemia time, typically considered to be within 20–30 min, is crucial to prevent irreversible damage to the renal parenchyma^2^.

Using 3D printing (3DP) technology in the medical field has been found to provide significant advantages in various areas such as patient-specific surgical guides, preoperative planning and simulation, educational phantom, and prosthetic fabrication^3,4^. The utilization of 3DP, also known as additive manufacturing or rapid prototyping, involves constructing objects by adding layers until it is completed, as opposed to subtractive manufacturing, which involves removal of materials. It offers the benefits of producing complex designs with high precision and cost and time efficiency^4,5^. Among the various applications of 3DP, the use of patient-specific 3DP surgical guides have seen significant advancements and further research is underway.

In breast surgery, patient-specific surgical guides are being used to aid in the removal of breast cancer during partial mastectomies by injecting blue dye and marking the location of the margin, including the area to be removed^6,7^. Similarly, the 3D printed surgical guides that can remove skin cancer including margins are applied in dermatology^8^. This enables accurate removal of the tumor while preserving healthy tissue and improvement for the surgical outcomes.

3DP simulators for partial nephrectomy, including renal cell carcinoma (RCC), are being used in various research studies in urology. Bernhard et al.^9^ developed patient-specific kidney tumor phantoms based on computed tomography (CT) images of seven patients who were considering partial nephrectomy. They compared the results before and after using the education-based phantoms and found that it improved the patients’ knowledge and understanding of basic kidney physiology, anatomy, tumor characteristics, and the planned surgical procedure. Similarly, Wake et al.^10^ used a 3DP simulator with RCC based on complex structures for pre-surgical planning in 10 patients. The 3D models lead to changes in decision making for transperitoneal or retroperitoneal approaches and clamping with a range of 30–50%. However, these two studies focused on education for patients and decision making for surgeon and not directly affect the removal of RCC in partial nephrectomy. Therefore, we developed patient-specific 3DP kidney surgical guide (3DP-KSG) that can remove RCC including margin and realistic phantoms using silicon to evaluate the accuracy of 3DP-KSG.

## Methods

### Overall procedure

**Figure 1:**
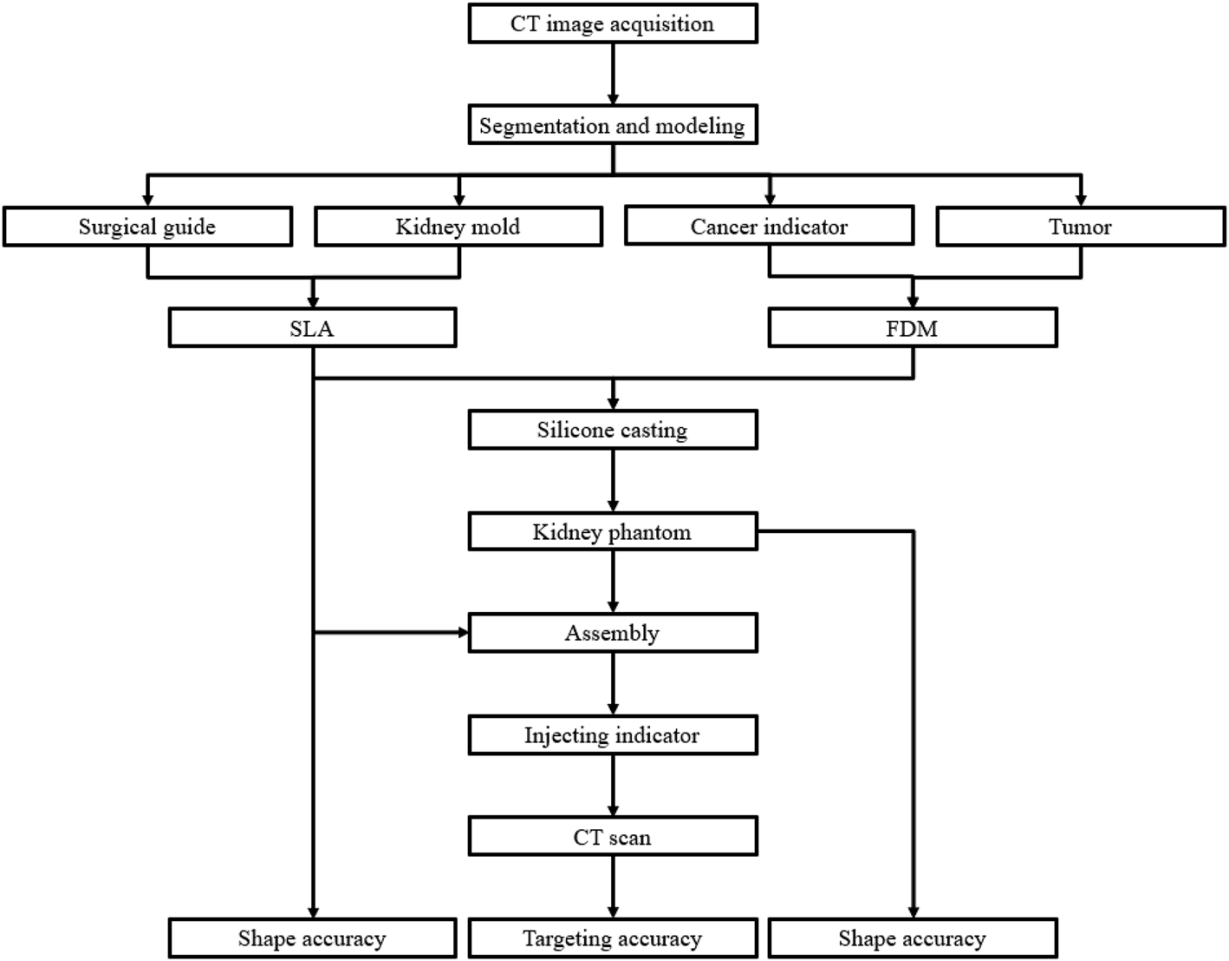
Overall procedure of fabricating and evaluating a 3D-printed kidney cancer resection guide and realistic kidney phantom. (CT = computed tomography, SLA = stereolithography apparatus, FDM = fused deposition modeling).

To quantitatively evaluate a patient-specific 3DP-KSG’s targeting accuracy, a realistic phantom and surgical guide are required, and it includes various steps and technologies such as 3DP technology, post-processing, and silicone casting technologies (Fig. 1).

### CT image acquisition and segmentation

**Figure 2:**
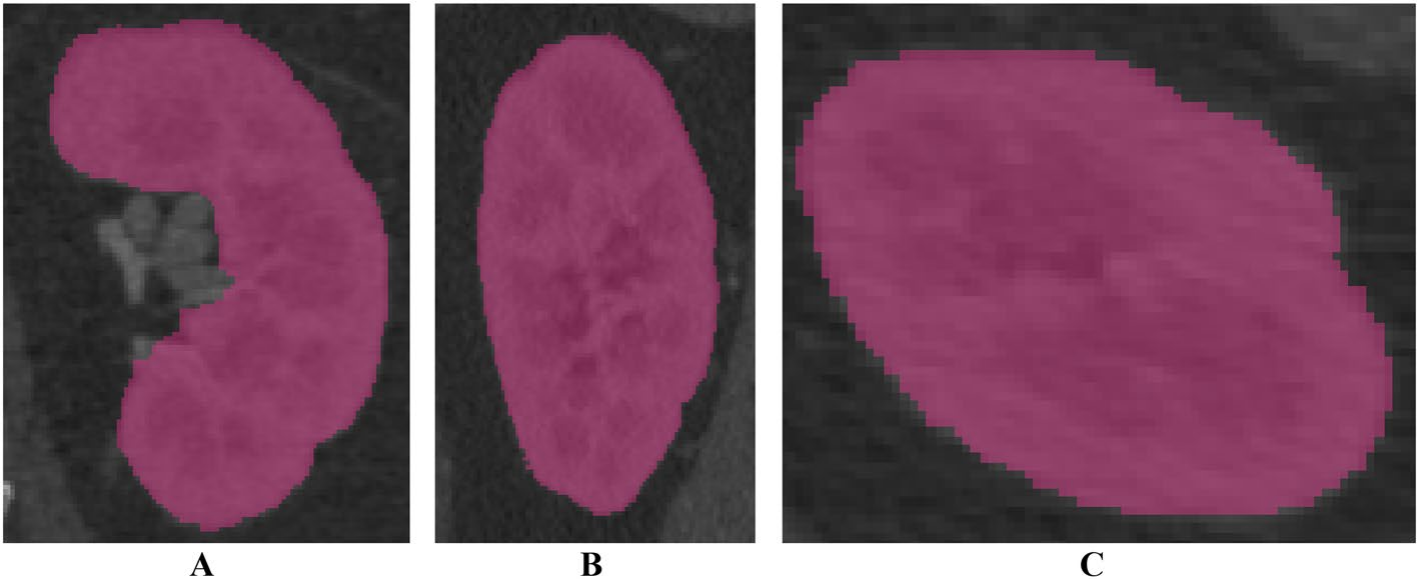
Visualization of the segmented left kidney in computed tomography images of a 47-year-old patient who was diagnosed with partial nephrectomy. The view of (**A**) coronal, (**B**) sagittal, and (**C**) axial.

This study was approved by the Institutional Review Board of Asan Medical Center (IRB No. 2021-0449) and conducted in accordance with the principles of the Declaration of Helsinki. Written informed consent was obtained from the patient. Abdominal multi-detector computed tomography (MDCT) scans (LightSpeed VCT, GE Healthcare, Chicago, USA) were obtained from a 47-year-old female scheduled for partial nephrectomy, using a slice thickness of 1.25 mm. The images were anonymized. The kidney parenchyma was segmented using Mimics ver. 17 (Materialise Inc., Leuven, Belgium) with thresholding functions of 123–1546 Hounsfield units (HU) and region growing functions using manually chosen seeds by an expert (Fig. 2). The segmented parenchyma was then converted to a kidney phantom model in stereolithography (STL) format.

### Modeling of kidney phantom

**Figure 3:**
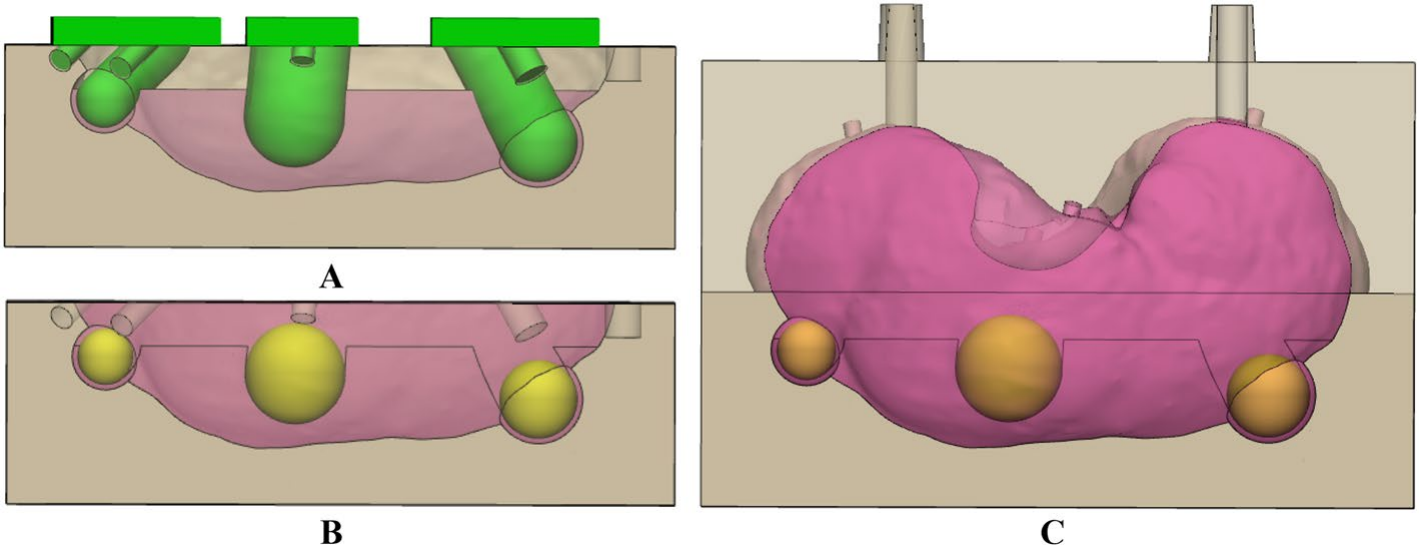
Procedure of pouring silicone into kidney molds. (**A**) One-third of the lower mold was filled with silicone. (**B**) Two-thirds was filled with silicone and the fabricated tumor was placed. (**C**) The upper and lower molds were combined, and silicone was injected completely.

The patient-specific kidney phantom consisted of the parenchyma and a tumor. The kidney mold and tumor were modeled using 3-matic ver. 9 (Materialise Inc., Leuven, Belgium). Only the left kidney was required to evaluate the 3DP-KSG; therefore, only one kidney was fabricated. The kidney mold was designed as a negative parenchyma-shaped body, which was divided into upper and lower sections. Furthermore, the lid was designed to indicate the location of the negative tumor shape. The tumor model was sphere shaped with radii of 5, 7, and 9-mm. Silicone was poured and casted into the kidney mold in three steps: First, silicone was poured up to one-third into the lower mold, and the lid was closed to create a negative-shaped tumor (Fig. 3A). Second, the fabricated tumor was placed on the negative-shaped tumor, and silicone was filled up to two-thirds (Fig. 3B). Finally, the upper and lower molds were combined, and silicone was injected to create the phantom (Fig. 3C). The kidney was removed from the mold by disassembling the upper and lower molds after allowing 1 day for the silicone to cure sufficiently.

### Modeling of kidney cancer resection guide

**Figure 4:**
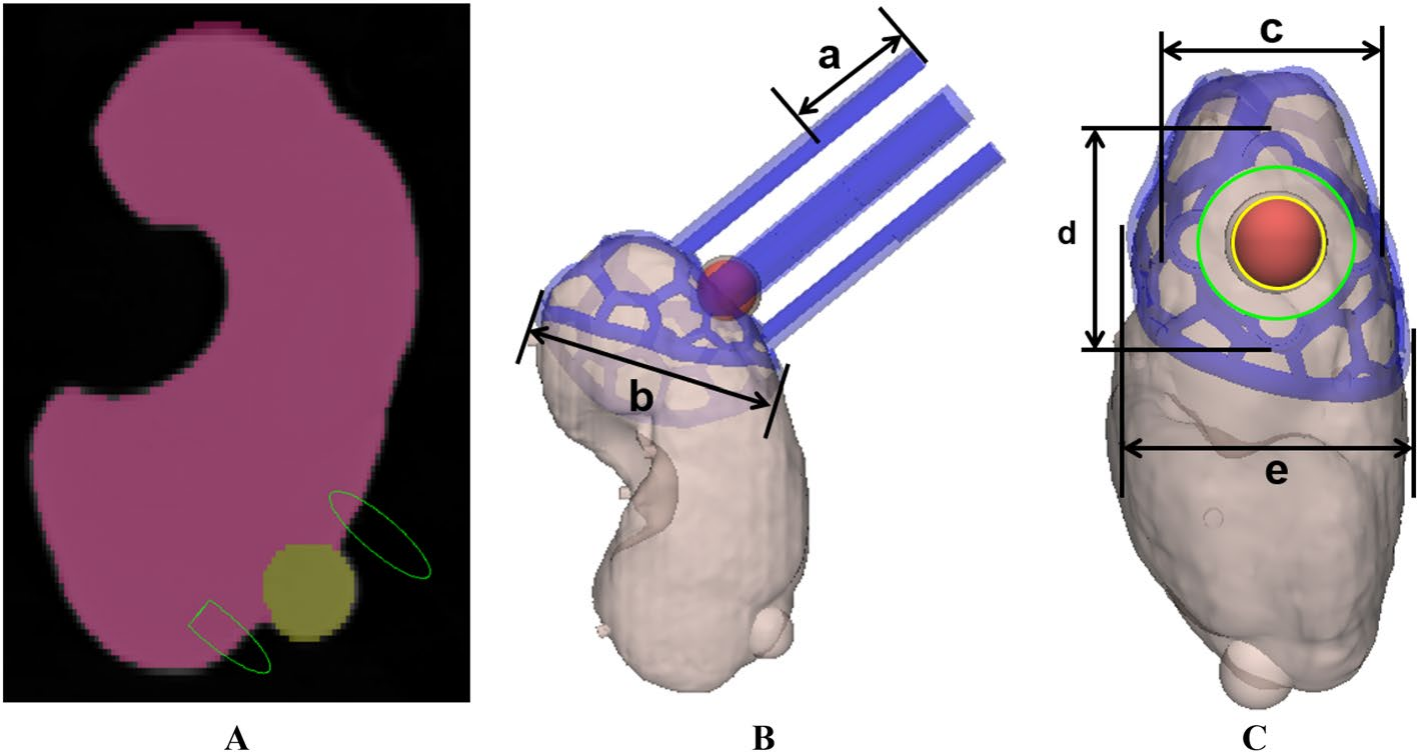
Visualization of the model kidney phantom for evaluating the 3D printing kidney cancer resection guide. (**A**) Coronal view of the segmented parenchyma and tumor and designated resection point and depth. (**B**) Visualization of the depth of the guide using the intravenous catheters and measurement point for evaluating fabrication accuracies; (a) height of the columns and (b) length of the guide. (**C**) Visualization of the tumor outline (yellow), safety margin (green), and measurement point for evaluating fabrication accuracies; (c) width of the columns, (d) length of the columns, and (e) width of the guide.

The 3DP-KSG was modeled using the segmented kidney and modeled tumor, and safety margin recommended by the surgeon (Fig. 4A). The safety resection margin of the tumor was set at 5 mm, and four insertion points, including the top, bottom, left, and right of the tumor, were designated based on the safety margin line (Fig. 4C). The 3DP-KSG’s columns were designed to insert the 16-gauge intravenous catheters into the end of the tumor, including the safety margin, at each point (Fig. 4B). Furthermore, the body of the 3DP-KSG was modeled to cover a portion of the parenchyma. The modeled body was porous to minimize the material consumption (Fig. 4B,C).

### Fabrication of kidney cancer phantom and resection guide

**Figure 5:**
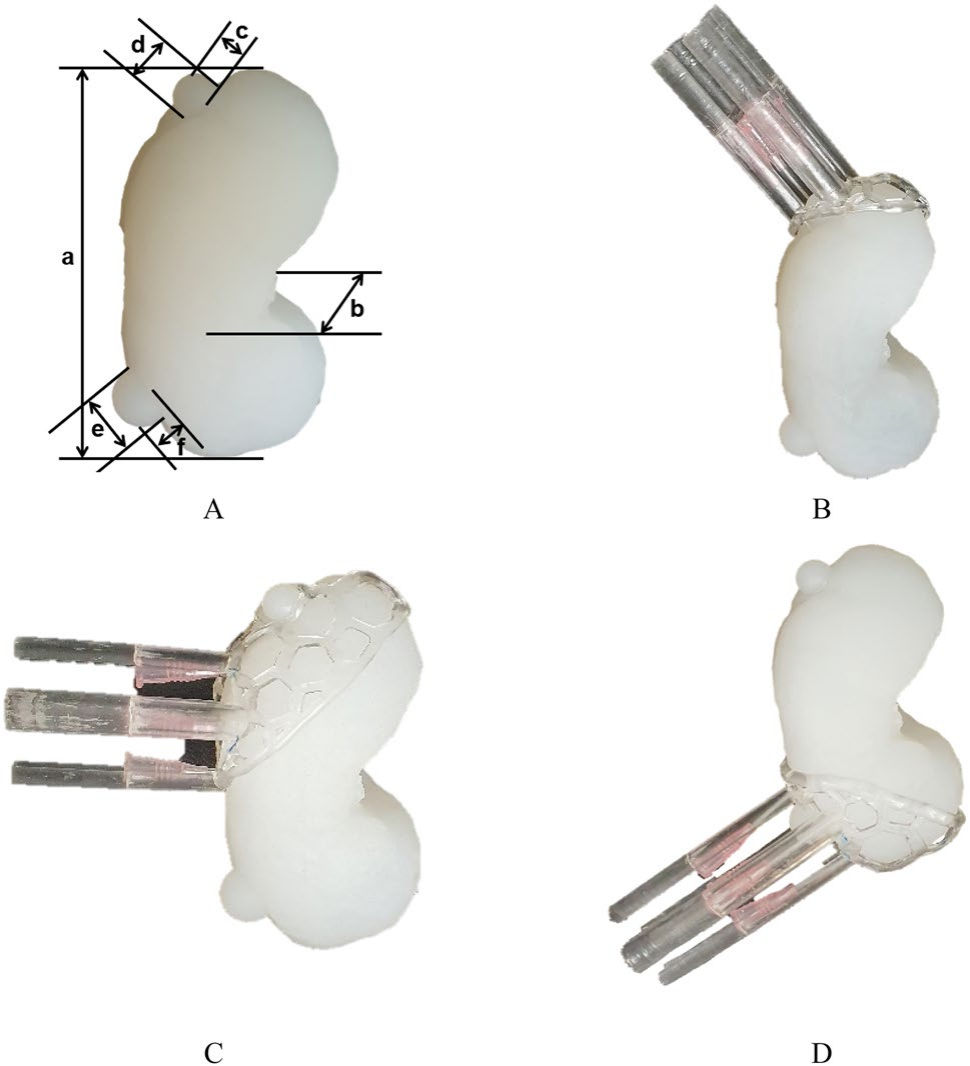
Fabricated kidney phantom and inserted 16-gauge intravenous (IV) catheter using the kidney cancer resection guide. (**A**) Fabricated kidney phantom through silicone casting and measurement points for evaluating fabrication accuracies; length (a) and width (b) of the phantom, length (c) and width (d) of the upper tumor, and length (e) and width (f) of the lower tumor. (**B**) Inserted catheters into the kidney phantom using 3D printing kidney surgical guide (3DP-KSG) attached to the upper tumor. (**C**) Inserted catheters into kidney phantom using 3D printing kidney surgical guide (3DP-KSG) attached to the middle tumor. (**D**) Inserted catheters into kidney phantom using 3D printing kidney surgical guide (3DP-KSG) attached to the lower tumor.

Each mold was fabricated using stereolithography apparatus (SLA) with clear resin (Form3, Formlabs, Massachusetts, USA) to ensure proper silicone spread, and the tumor was also fabricated using fused deposition modeling (FDM) with polylactic acid (PLA) filament (DP200, Sindoh, South Korea). The lid was fabricated using FDM with PLA filament due to its adequate hardness and cost effectiveness. The elastic modulus of the human kidney is 180.32 kPa, which is similar to a 170 kPa of ecoflex00-30 silicone^11,12^. Therefore, the kidney parenchyma was fabricated using silicone (Ecoflex00-30, Smooth-On, USA) (Fig. 5A). Finally, the 3DP-KSG was fabricated using SLA with dental surgical guide resin (Form3, Formlabs, Massachusetts, USA) (Fig. 5B–D).

### Targeting and shape accuracy

The fabrication accuracy of the 3DP-KSG and kidney phantom should be prioritized before measuring the targeting accuracy of the 3DP-KSG. Therefore, we evaluated shape accuracy by measuring phantom and 3DP-KSG. Three operators measured the thickness of the width, length, and height of the phantom and 3DP-KSG of the STL models and fabricated models five times using 3-matic software and the vernier calipers (Figs. 4B,C, and 5A).

In addition, three operators independently inserted catheters into the kidney phantom three times, after receiving pre-training on how to use the 3DP-KSG. The phantom with inserted catheters was scanned using MDCT (SOMATOM Definition Flash, SIEMENS, Munich, Germany) using a slice thickness of 1 mm. The parenchyma and catheters were roughly segmented using thresholding functions (− 178 to 3070 HU and − 900 to 3070 HU, respectively), and specific objects were selected using the region-growing function. The segmented parenchyma and catheters were then converted to STL models and matched using the global registration function with manual correction applied to the planned STL model. A total of 108 points were derived from the STL models, and the differences in planned and actual points and trajectories were measured using 3-matic.

### Statistical analysis

A Bland–Altman analysis was used to evaluate the planned and actual points using Med-Calc ver. 19 (MedCalc Software Ltd., Acacialaan, Belgium). The fabrication accuracies of the phantom and 3DP-KSG were also analyzed using Bland–Altman analysis. The intraclass correlation coefficient (ICC) was used to compare significant differences among the operators using IBM SPSS Statistics v25.00 (IBM Corp., New York, USA). The ICC value was used to represent the level of precision at a 95% confidence interval.

## Results

### Accuracy evaluation of phantom and guide

**Figure 6:**
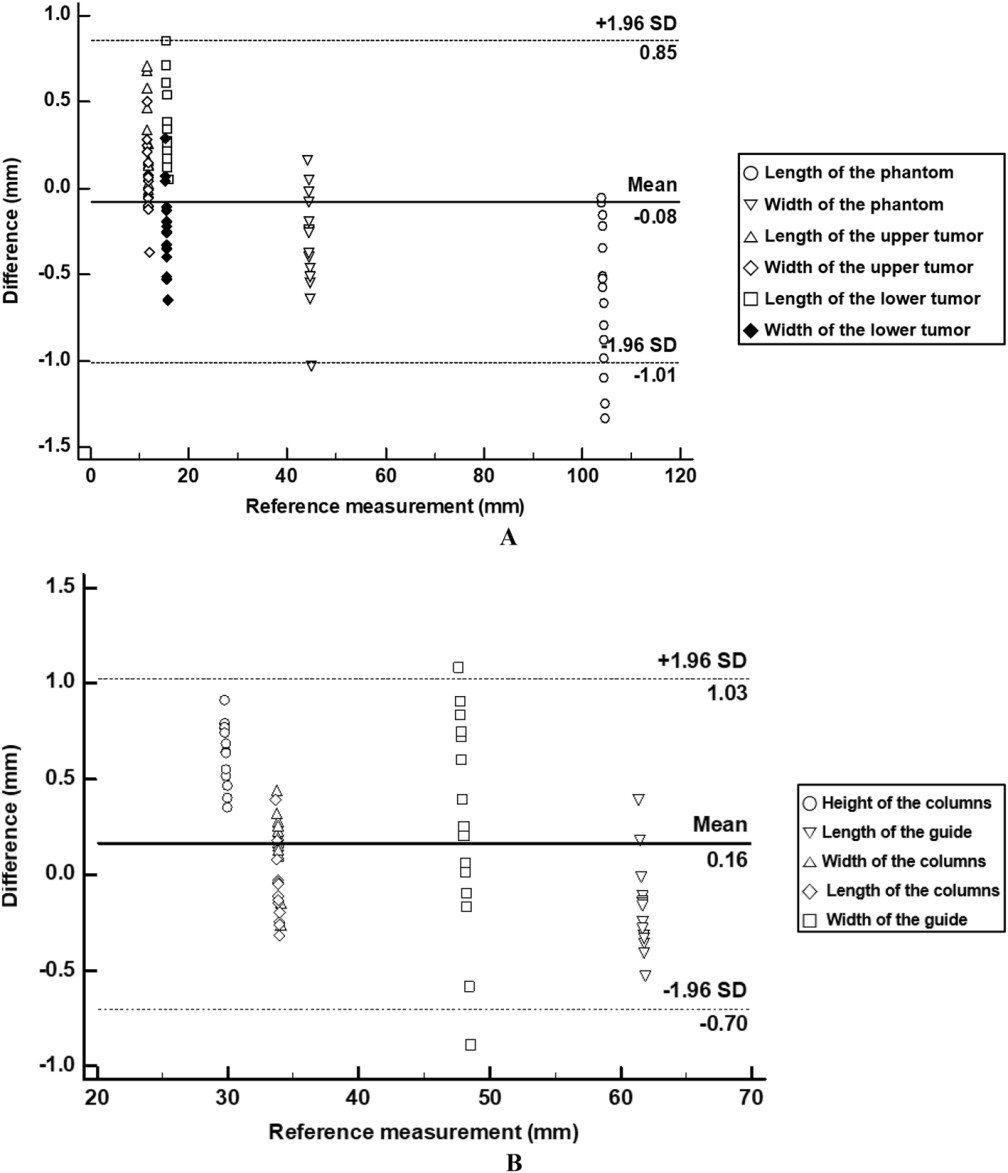
Comparison of stereolithography (STL) models and fabricated models with (**A**) Bland–Altman plot to evaluate differences between the STL models and fabricated kidney phantom, and (**B**) Bland–Altman plot to evaluate differences between the STL models and fabricated kidney surgical guide.

We evaluated the measurement error between the STL models and fabricated models in kidney phantom and 3DP-KSG using the Bland–Altman plot. The measurement error of the kidney phantom (mean ± SD) was 0.35 ± 0.29 mm (limit of agreement from − 1.01 to 0.85 mm) (Fig. 6A). All measurements, except for a few of the length and width of the phantom, were within the 95% limit of agreement. The measurement error of 3DP-KSG (mean ± SD) was 0.36 ± 0.26 mm (limit of agreement from − 0.70 to 1.03 mm) (Fig. 6B) All measurements, except two of the width of the guide, were within the 95% limit of agreement.

### Evaluation of injection point

**Figure 7:**
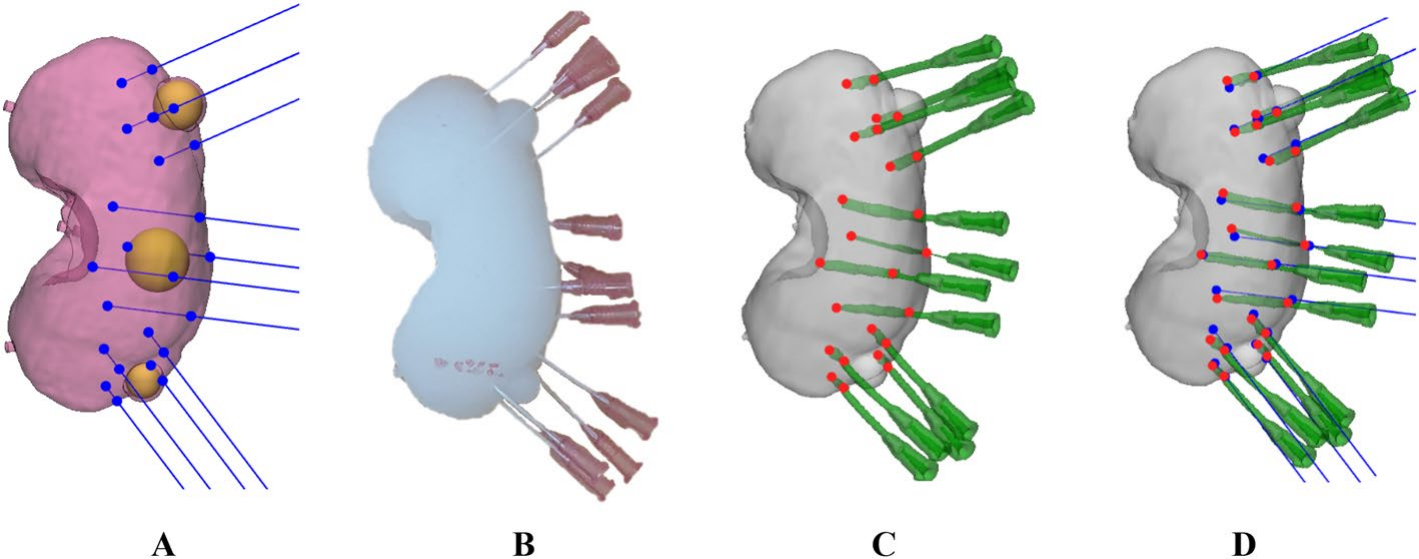
Visualization of the kidney phantom with an inserted 16-gauge intravenous (IV) catheter using 3D printing kidney surgical guide (3DP-KSG). (**A**) Planned insertion points and lines. (**B**) Inserted 16-gauge IV catheters into the kidney phantom using 3DP-KSG. (**C**) Actual insertion points of the catheter. (**D**) Matching planned points with actual inserted points.

**Figure 8:**
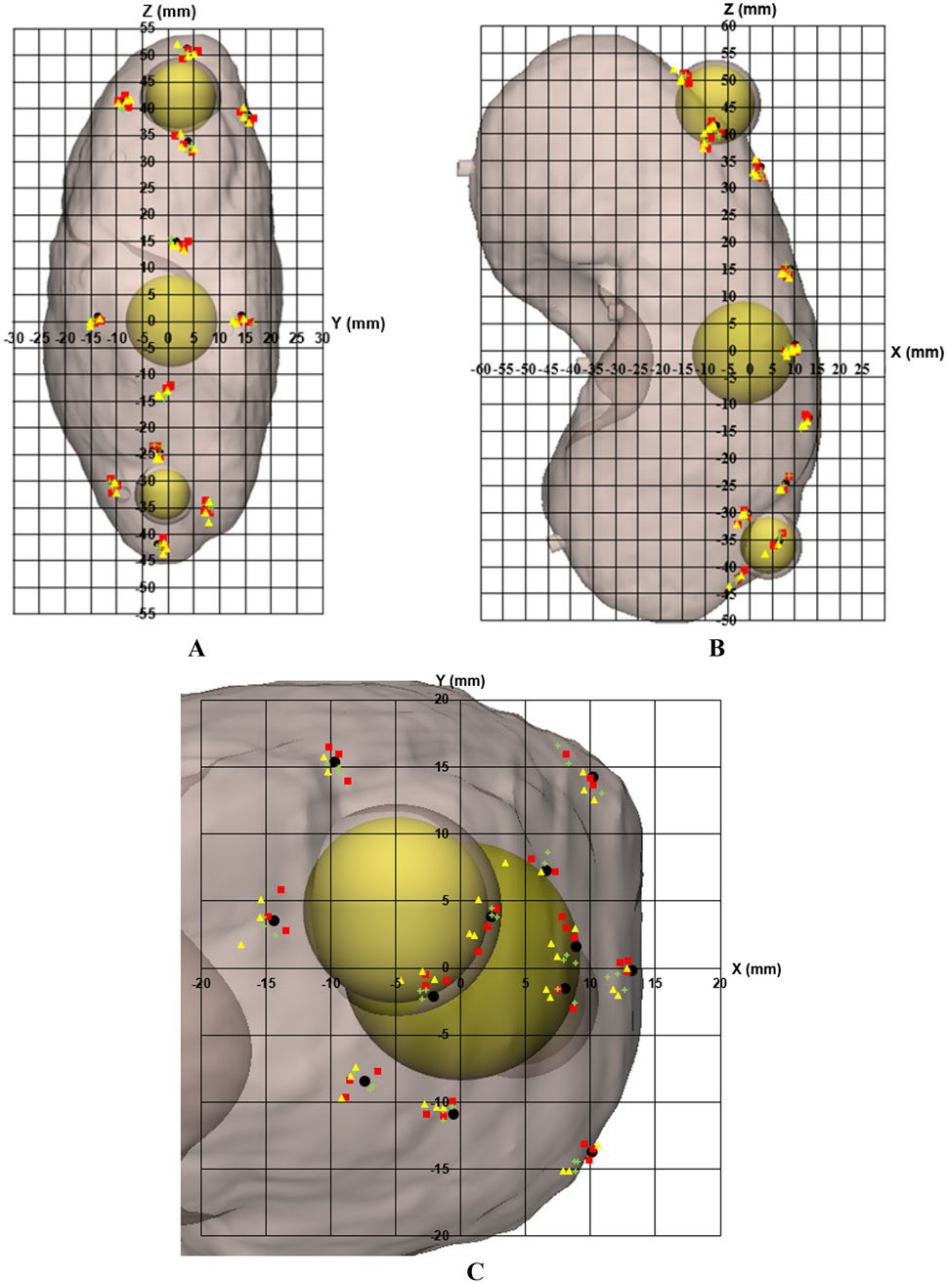
Dot plot of the planned and actual insertion point placement on the kidney at the origin of the center of the middle tumor (0, 0), and (**A**) sagittal (Y–Z), (**B**) coronal (X–Z), and (**C**) axial (X–Y) views were visualized. X- (left to right), Y- (anterior to posterior), and Z- (superior to inferior). (Reference point, black dot; observer 1, yellow triangle; observer 2, green plus sign; observer 3, red rectangle).

**Figure 9:**
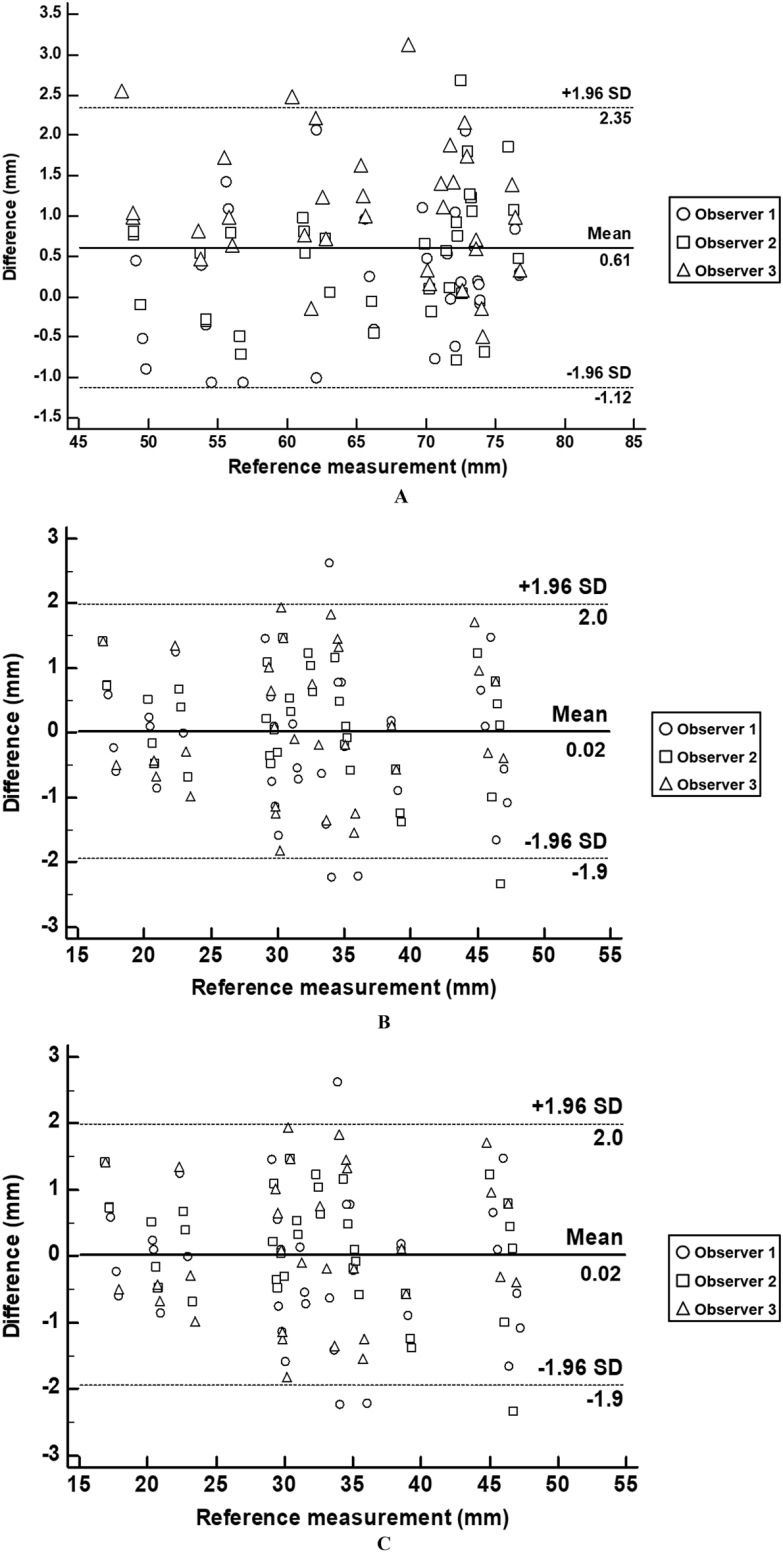
Bland–Altman plot evaluation of the accuracy of the entry point of 3D printing kidney surgical guide for (**A**) X, (**B**) Y, and (**C**) Z axes. X- (left to right), Y- (anterior to posterior), and Z- (superior to inferior).

We extracted a total of 108 insertion points from the STL models and compared them with the planned points (Fig. 7). The measurement error of insertion point (mean ± standard deviation (SD)) was 1.60 ± 0.74 mm. In addition, we created a line connecting the entry points and the endpoints to extract vectors and measured the cosine similarity between the vector of the planned line and the measured line, which was 0.99 ± 0.01. Figure 8 illustrates a graphic representation of the planned and actual points on the kidney surface in the coronal, sagittal, and axial views. Furthermore, the Bland–Altman plot was used to evaluate the accuracy of the entry point of the 3DP-KSG for the X, Y, and Z-axes. The measurement error (mean ± SD) for each axis was 0.61 ± 0.86 mm (limit of agreement from − 1.12 to 2.35 mm), 0.03 ± 1.00 mm (limit of agreement from − 1.9 to 2.0 mm), and − 0.51 ± 0.92 mm (limit of agreement from − 1.30 to 2.32 mm) (Fig. 9.). The statistical differences between operators were analyzed using the ICC. The ICC for each axis was 0.957 (X-axis), 0.981 (Y-axis), and 0.999 (Z-axis).

## Discussion

When performing a partial nephrectomy, warm ischemia time should be kept to an absolute minimum which requires the surgeon to advance through the surgery fluently^2^. The 3DP-KSG is designed to be fixed in a form that wraps around a portion of the kidney and can accurately mark the resection area with a safety margin on the kidney before vessels are clamped. This suggests that the procedure can be performed more accurately and quickly. We fabricated a realistic kidney phantom with similar elastic modulus and accurate shape to measure the targeting accuracy of the 3DP-KSG. This will provide a realistic simulation of a partial nephrectomy. Using a Bland–Altman plot to measure the fabrication accuracy of the kidney phantom demonstrated a reasonable error (limit of agreement ranging from − 1.01 to 0.85 mm). A slightly increased error was noted in measuring the length of the phantom, which may be due to the relatively large measurement range and high flexibility and is unlikely to impact its usability, as the difference remained within 1 mm. Similarly, the 3DP-KSG was also evaluated for fabrication accuracy using a Bland–Altman plot, which showed stable errors (limit of agreement ranged from − 0.70 to 1.03). The difference was < 1 mm indicating no significant issue in its use, although the width of the guide displayed a relatively high error. We also evaluated the accuracy of insertion points using the 3DP-KSG with the realistic kidney phantom. We measured the accuracy of the insertion point on the kidney surface because the area was marked directly on the kidney surface when performing surgery using 3DP-KSG. The insertion point measurement error was reasonable at 1.60 ± 0.74 mm. Furthermore, each measurement error measured in the X, Y, and Z axes was reasonable at 0.61 ± 0.86, 0.03 ± 1.00, and − 0.51 ± 0.92 mm, respectively. In addition, the most important part of the surgical guide was to minimize the variation between users. Therefore, the ICC of each axis for evaluating statistical differences between the three operators was 0.957 (X-axis), 0.981 (Y-axis), and 0.999 (Z-axis), which shows excellent inter-rater agreement. These results show that anyone can obtain accurate and identical surgical results using 3DP-KGS. Accuracy using 3DP-surgical guide is around 1–2 mm in case of dental implant placement^13,14^. Our 3DP-KSG showed similar targeting accuracy of dental implant guide for hard tissue. In our study, the phantom was fabricated through silicone casting, considering the mechanical properties of an actual kidney. In a real environment, it would be more cost-effective and check the accuracy of 3DP-KSG by fabricating a modeled kidney phantom with a hole of a resection point using FDM and thermoplastic polyurethane filament.

This study has several limitations. First, we were unable to evaluate targeting accuracy for multiple tumors. Follow-up studies will be conducted to evaluate improved 3DP-KSG using the kidney phantom for tumors of larger and wider distributions. Second, a simulation environment with similar mechanical properties of the kidney was proposed. However, the simulation environment is not similar to an actual surgical environment with a limited field of vision of the actual procedure site. These limited environments could reduce the accuracy of 3DP-KSG. For future research, we will implement more realistic phantoms to measure targeting accuracy by not only fabricating kidney phantoms using silicon but also incorporating surrounding structures such as organs, bones, fat, skin, and blood vessels to establish conditions similar to the actual surgical environment. Third, we used only one SLA with dental SG resin to evaluate the 3DP-KSG, which can result in relatively low reproducibility. Follow-up studies will be conducted using various kinds of SLA printers and resins. Forth, in this study, only medical 3DP experts participated in the experiment. The accuracy could be decreased if knowledge regarding 3DP technology and medical imaging is lacking. Therefore, Follow-up studies will consider the participation of various groups such as various levels of surgeons. Fifth, 3DP-KSG did not compare with existing technologies. In partial nephrectomy, surgeons determine the resection line using an ultrasonography scan in real time. However, our kidney phantoms cannot detect tumors by ultrasonography. In a follow-up study, the kidney phantom will be fabricated tumor can be detected using ultrasound, and the resection line created using ultrasound and 3DP-KSG will be compared. Finally, 3DP-KSG was developed with a specific focus on open partial nephrectomy, and its morphological structure renders it unsuitable for application in robot-assisted or laparoscopic partial nephrectomy. Therefore, in future research, we plan to incorporate elastic 3D printing and 4D printing technologies using shape memory polymer materials.

## Conclusion

The 3DP-KSG demonstrated precise and quantitative targeting of the kidney tumor in a consistent manner with no significant variability between operators. The surgeon could effectively and rapidly remove the lesion with accuracy during partial nephrectomy using the 3DP-KSG.

## Data Availability

The datasets generated in this study during the current study are not publicly available because the data used in our study were created based on patient images but are available from the corresponding author on a reasonable request.

## Abbreviations

3DP: 3D printing
CT: Computed tomography
3DP-KSG: 3D-printed kidney surgical guide
ICC: Intraclass correlation coefficient
SLA: Stereolithography apparatus
FDM: Fused deposition modeling
PLA: Polylactic acid
MDCT: Multi-detector computed tomography
STL: Stereolithography
